# The impact of shift work induced chronic circadian disruption on IL-6 and TNF-α immune responses

**DOI:** 10.1186/1745-6673-5-18

**Published:** 2010-07-05

**Authors:** Anke van Mark, Stephan W Weiler, Marcel Schröder, Andreas Otto, Kamila Jauch-Chara, David A Groneberg, Michael Spallek, Richard Kessel, Barbara Kalsdorf

**Affiliations:** 1 Institute of Occupational Medicine, University of Lübeck, 23538 Lübeck, Ratzeburger Allee 160, Germany; 2 AUDI AG Ingolstadt, 85045 Ingolstadt, Germany; 3 Department of Psychiatry and Psychotherapy, University of Lübeck, Ratzeburger Allee 160, 23538 Lübeck, Germany; 4 Institute of Occupational Medicine, Charité - Universitätsmedizin Berlin, Free University and Humboldt-University, 14195 Berlin, Thielallee 73, Germany; 5 European Research Society for Environment and Health in Transportation (EUGT) e. V., 14195 Berlin, Thielallee 73, Germany; 6 Clinical Infectious Diseases, Research Center Borstel, 23845 Borstel, Germany

## Abstract

**AIM:**

Sleep disturbances induce proinflammatory immune responses, which might increase cardiovascular disease risk. So far the effects of acute sleep deprivation and chronic sleep illnesses on the immune system have been investigated. The particular impact of shift work induced chronic circadian disruption on specific immune responses has not been addressed so far.

**Methods:**

Pittsburgh-Sleep-Quality-Index (PSQI) questionnaire and blood sampling was performed by 225 shift workers and 137 daytime workers. As possible markers the proinflammatory cytokines IL-6 and TNF-α and lymphocyte cell count were investigated. A medical examination was performed and biometrical data including age, gender, height, weight, waist and hip circumference and smoking habits were collected by a structured interview.

**Results:**

Shift workers had a significantly higher mean PSQI score than day workers (6.73 vs. 4.66; p < 0.001). Day workers and shift workers had similar serum levels of IL-6 (2.30 vs. 2.67 resp.; p = 0.276), TNF-α (5.58 vs. 5.68, resp.; p = 0.841) or lymphocytes count (33.68 vs. 32.99, resp.; p = 0.404). Furthermore there were no differences in cytokine levels (IL-6 p = 0.761; TNF-α p = 0.759) or lymphocyte count (p = 0.593) comparing the sleep quality within the cohorts. When this calculation of sleep quality was stratified by shift and day workers irrespective of their sleep quality day workers and shift workers had similar serum levels of IL-6, TNF-α or lymphocytes count. Multiple linear regression analysis showed a significant correlation of lymphocytes count and smoking habits.

**Conclusion:**

Shift work induces chronic sleep debt. Our data reveals that chronic sleep debt might not always lead to an activation of the immune system, as we did not observe differences in lymphocyte count or level of IL-6 or TNF-α serum concentration between shift workers and day workers. Therefore chronic sleep restriction might be eased by a long-term compensating immune regulation which (in healthy) protects against an overstimulation of proinflammatory immune mechanisms and moderates metabolic changes, as they are known from short-term sleep deprivation or sleep related breathing disorders.

## Introduction

Shift-workers are forced to work and sleep against normal chronobiological rhythms: they sleep at times their organism is set to activity and they work when physical and psychical effectiveness is generally low. These contradictious demands induce various indispositions; most frequently sleep disturbances which can cause severe sleep debt [1-6]. Recent research focuses on investigations how these sleep disturbances influence the immune system and if such an activation of the immune system might be linked with cardiovascular disease risks.

Activation of inflammatory immune responses is marked by an increase of proinflammatory cytokines, e.g. Interleukin-1beta (IL-1ß), IL-6 or Tumor-Necrosis-Factor alpha (TNF-α). Several authors report an augmentation of these cytokines after acute sleep deprivation and thereby an induction of proinflammatory immune responses [7-9]. Cytokine levels correlate with fatique and daytime somnolence [10-12]. Therefore Vgontzas *et al. *call IL-6 and TNF-α fatique inducing cytokines [12]. Similar to acute sleep disorders people with a chronic sleep debt due to their obstructive sleep apnoe syndrome show elevated cytokine activity [13-16]. Cytokines regulate cell proliferation and differentiation. Thereby proinflammatory cytokine activation induces the recruitment of lymphocytes and neutrophils. Liu *et al. *demonstrated an increase of lymphocyte and neutrophil cells after acute sleep deprivation [17].

As inflammatory processes are recognized to play a major role in the pathogenesis of atherosclerosis, serum biomarkers are investigated to estimate emerging cardiovascular risk. There is increasing evidence that proinflammatory conditions and sleep disturbances elevate the risk of cerebrovascular and cardiovascular diseases [13,18-25]. This model may explain the higher cardiovascular diseases risk in shift workers [2,26-28].

So far this knowledge of associations between sleep debt and immune responses has not been related to the socioeconomically important factor shift work. We hypothesize that shift work induced chronic circadian disruption does affect proinflammatory immune responses. As possible markers of these changes in the immune system we investigated the proinflammatory cytokines IL-6 and TNF-α and measured the lymphocyte cell count.

## Materials and methods

The study was approved by the Research Ethics Committee of the University Schleswig-Holstein Campus Lübeck, Germany (Ref. Az 05-028). Following informed consent, peripheral venous blood was drawn and the Pittsburgh-Sleep-Quality-Index (PSQI) questionnaire was completed by 225 shift workers and 137 daytime workers. A medical examination was performed and biometrical data including age, gender, height, weight, waist and hip circumference and smoking habits were collected by a structured interview. Both cohorts had a similar social background and worked in the industrial sector. Most shift workers worked in a three shift system including night shifts (91%), 9% worked in other schedules like permanent night shift, 24-hours or similarly irregular shift schedules.

The mean age of shift workers was 36.4 yrs (SD 9.34) and the mean age of daytime workers 40.1 yrs (SD 7.77; p < 0.001). 86.9% of the shift workers and 75.7% of the day workers were male (p = 0.010). Shift workers and day workers did not differ in their Body Mass Index (BMI; kg/m^2^), (mean 26.62 versus 26.27 respectively, p = 0.455). Shift workers were significantly more smokers than daytime workers (41.8% vs. 27.0%; p = 0.005).

The PSQI is a validated instrument composed of 19 self-rated questions for the measurement of sleep quality during the previous month. Higher PSQI-Sum-Scores indicate inferior sleep quality. The questionnaire divides into "good sleeper" (PSQI ≤ 6), "poor sleeper" (PSQI 6 - 10) and "people with chronic sleep disorders" (PSQI ≥ 11), but the test does not enable to distinguish between different causes of sleep disorders. For this study a total value of 6 was defined the limit for the diagnosis "disturbed sleep", meaning values ≥ 6 are a proved sign for relevant sleep disturbances, values < 6 signify none or slight, clinically not relevant sleep disturbances.

Fasting blood samples were withdrawn between 6 to 8 am, promptly centrifugated and aliquots stored at -40°C until analysis. Concentration of IL-6 and TNF-α were measured by ELISA-techniques as recommended by the manufacturer (Immulite, Los Angeles, Germany). Blood cell count was detected immediately after sampling with an automated hematology analyzer.

Statistical test were performed with descriptive methods, Levene's test for equality of variances, and t-tests for mean comparisons if applicable. Independent dichotomous variables were analyzed by Chi-Square-test, metric data by t-test, correlation coefficients were calculated on a ranked basis by spearman-rho procedure. Significance level was defined as p < 0.05. Data were normally distributed. Missing data rate was below 6%.

## Results

Shift workers had a significantly higher mean PSQI score than day workers (6.73 vs. 4.66; p < 0.001) and significantly more shift workers had pathologically elevated scores (61.2% vs. 26.7%; p < 0.001).

Day workers and shift workers had similar serum levels of IL-6 (p = 0.276), TNF-α (p = 0.841) or lymphocytes count (p = 0.404) (table 1). Furthermore there were no differences in cytokine levels (IL-6 p = 0.761; TNF-α p = 0.759) or lymphocyte count (p = 0.593) within the cohorts comparing their sleep quality (table 1). Neither when this calculations of sleep quality was stratified by shift and day workers, i.e. example, IL-6 concentration in day workers with good sleep quality was 2.31 μg/ml in comparison to 2.67 μg/ml IL-6 in shift workers with good sleep quality (table 2). Comparing IL-6 concentration in respect to sleep quality shift workers with good sleep quality had a similar IL-6 levels (2.67 μg/ml) as their shift working colleges with relevant sleep disturbances (2.72 μg/ml) (table 2).

**Table 1:** Influence of shift work and sleep quality by PSQI on IL-6, TNF-α and lymphocytes.

*Component *	*Collective *	*Mean *	*SD *	*p *
IL-6	Daytime workers Shift workers	2.30 2.67	1.54 3.79	0.276
TNF-α	Daytime workers Shift workers	5.58 5.68	2.91 5.19	0.841
Lymphocytes	Daytime workers Shift workers	33.68 32.99	7.64 7.49	0.404

***Component ***	***PSQI ***	***Mean ***	***SD ***	***p ***
IL-6	PSQI ≤ 6 PSQI ≥ 6	2.47 2.58	2.50 3.77	0.761
TNF-α	PSQI ≤ 6 PSQI ≥ 6	5.53 5.68	3.49 5.41	0.759
Lymphocytes	PSQI ≤ 6 PSQI ≥ 6	33.59 33.14	7.52 7.78	0.593

**Table 2:** Influence of sleep quality by PSQI on IL-6, TNF-α and lymphocytes stratified by shift and daytime worker.

*Sleep quality*	*Component*	*Collective*	*Mean*	*SD*	*p*
PSQI < 6	IL-6	Daytime workers Shift workers	2.31 2.67	1.59 3.31	0.357
	TNF-α	Daytime workers Shift workers	5.51 5.55	3.01 4.02	0.945
	Lymphocytes	Daytime workers Shift workers	33.98 33.12	7.60 7.44	0.453

PSQI ≥ 6	IL-6	Daytime workers Shift workers	2.06 2.72	0.31 4.24	0.362
	TNF-α	Daytime workers Shift workers	5.54 5.73	2.06 6.07	0.853
	Lymphocytes	Daytime workers Shift workers	32.85 33.17	8.26 7.73	0.834

Multiple linear regression analysis showed a significant correlation of lymphocytes count and smoking habits. In contrast lymphocyte count and BMI, age, sleep quality or shift working status did not correlate. And there was no correlation of IL-6 or TNF-α with any of the above mentioned parameters (table 3).

**Table 3:** Standardized correlation coefficient (ß) with IL-6, TNF-α and lymphocytes in multiple regression analysis

*Component*	*Factor*	*ß*	*t*	*p*
IL-6	BMI	0.103	1.842	0.066
	Shift work	0.049	0.820	0.413
	Age	0.006	0.103	0.918
	Smoking	0.100	1.794	0.074
	sleep quality	0.006	0.099	0.921

TNF-α	BMI	0.025	0.447	0.656
	Shift work	0.010	0.168	0.866
	Age	- 0.016	- 0.275	0.783
	Smoking	- 0.022	- 0.400	0.690
	sleep quality	0.014	0.239	0.811

Lymphocytes	BMI	- 0.043	- 0.771	0.441
	Shift work	- 0.005	- 0.088	0.930
	Age	- 0.068	- 1.208	0.228
	Smoking	- 0.170	- 3.092	0.002
	sleep quality	- 0.035	- 0.606	0.545

In a last step cytokine levels were correlated with blood cell counts. Linear regression analysis revealed no significant correlation of the cytokines and monocytes count (IL-6: ß = 0.008; T = 0.153; p = 0.879 or TNF-α: ß = - 0.025; T = - 0.475; p = 0.635) and neutrophils count (IL-6: ß = 0.034; T = - 0.542; p = 0,588; TNF-α: ß = - 0.021; T = - 0.343; p = 0.732) as well as lymphocytes count (IL-6: ß = - 0.002; T = - 0.036; p = 0.971; TNF- α: ß = 0.014; T = 0.256; p = 0.798).

## Discussion

The PSQI was used to evaluate sleep quality of the participants. Shift workers reported significantly worsened sleep quality compared to day workers. This is in line with many other authors who have demonstrated that shift work induces sleep disturbances and chronic sleep debt [1-6].

To investigate the long-term influence of chronic sleep debt and circadian disruption on the immune system we compared cytokine production and lymphocytes count in a morning blood sample of shift workers with daytime workers. Our results did not indicate an influence of shift work on cytokine levels of IL-6 or TNF-α and lymphocyte cell count. Furthermore there was no association between sleep quality and these indicators of inflammation in this study.

Referring to cell counts Liu *et al. *reported a short-term effect of a one night sleep deprivation on white blood cells and neutrophils, which we cannot find in our study setting looking for long-term effects [17].

Similarly, literature results on cytokine production are contradictive. E.g. Patel *et al. *described a direct association between the length of sleep and the increase of IL-6 serum levels or the decline of TNF-α [7]. In contrast Prather *et al. *showed that self-reported higher sleep debt scores predict elevated cytokine-levels of IL-1ß and IL-6 [8]. As well Okun *et al. *found an association between bad sleep quality of pregnant women and elevation of IL-6 levels but no changes in TNF-α [29]. In their subsequent study in young healthy women IL-6 and TNF-α were not related with PSQI score and sleep duration, consistent with our data [30].

In another approach Vgontzas *et al. *reported short-terms effects of a one night-sleep deprivation, which changed the circadian pattern of IL-6 secretion [11]. And they observed similar effects in diseased people with chronic insomnia [12]. These results have to evoke the considerations that we might have missed possible differences as we have only investigated a single time point and no daytime secretion.

Our results support an association of IL-6 levels and smoking. Literature reveals smoking to be an important confounding factor for proinflammatory reaction and cardiovascular diseases [28,31]. Shift workers smoke more frequently independently from demographic factors as age, gender or education [26,32-34]. Even after adjusting shift workers and day workers in regards to the variables of age, socioeconomic status, physical activity, smoking, and occupational load the shift workers keep their elevated risk for cardiovascular diseases [35].

An association between shift work, BMI and cardiovascular risks can be suspected [26,28,32]. Therefore one would assume that higher BMI scores predict higher proinflammatory cytokines. Our data do not show any association between sleep disorders and BMI.

## Conclusion

In summary, shift workers experience significantly more sleep disorders than day workers. Shift work induces chronic sleep debt. Our data reveals that chronic sleep debt might not always lead to an activation of the immune system, as we did not observe differences in lymphocyte count or level of IL-6 or TNF-α serum concentration between shift workers and day workers. Therefore chronic sleep restriction might be eased by a long-term compensating immune regulation which (in healthy) protects against an overstimulation of proinflammatory immune mechanisms and moderates metabolic changes, as they are known from short-term sleep deprivation or sleep related breathing disorders. We can assume that long-term sleep debt in healthy may not lead to persistent proinflammatory changes in the immune system and to a consecutively higher risk for cardiovascular diseases. Further investigations are required to clarify the role of sleep in the immune system. Thus, most likely the increased cardiovascular disease risk of shift workers is caused from various parameters [28].

## Competing interests

The authors declare that they have no competing interests.

## Authors' contributions

AvM conceived of the study and its design, led the coordination, data collection, data analysis and interpretation of results and drafted the manuscript. SWW participated in the design of the study, data collection, data analysis and interpretation and in performing the statistical analysis. MSchroeder participated in the design of the study, study coordination, data collection, data analysis and interpretation and in performing the statistical analysis and took part in preparation of the manuscript. AO participated in the data collection, study coordination, data analysis and interpretation and in performing the statistical analysis and took part in preparation of the manuscript. KJC participated in the design of the study, data collection, data analysis and interpretation and took part in preparation of the manuscript. DAG participated in data interpretation and took part in preparation of the manuscript. MSpallek participated in the design of the study, study coordination and data collection. RK participated in the design of the study, study coordination, data interpretation and in preparation of the manuscript. BK participated in the design of the study, data analysis and interpretation and in drafting the manuscript. All authors read and approved the final manuscript.
